# Reliability of the mean flow index (Mx) for assessing cerebral autoregulation in healthy volunteers

**DOI:** 10.14814/phy2.14923

**Published:** 2021-06-26

**Authors:** Markus H. Olsen, Christian G. Riberholt, Ronni R. Plovsing, Kirsten Møller, Ronan M. G. Berg

**Affiliations:** ^1^ Department of Neuroanaesthesiology Copenhagen University Hospital Rigshospitalet Denmark; ^2^ Department of Neurorehabilitation / Traumatic Brain Injury Unit Copenhagen University Hospital Rigshospitalet Denmark; ^3^ Department of Anaesthesia Hvidovre Hospital University of Copenhagen Copenhagen Denmark; ^4^ Institute of Clinical Medicine Faculty of Health and Medical Sciences University of Copenhagen Copenhagen Denmark; ^5^ Department of Clinical Physiology, Nuclear Medicine & PET Copenhagen University Hospital Rigshospitalet Denmark; ^6^ Centre for Physical Activity Research Copenhagen University Hospital Rigshospitalet Denmark; ^7^ Department of Biomedical Sciences Faculty of Health and Medical Sciences University of Copenhagen Copenhagen Denmark; ^8^ Neurovascular Research Laboratory Faculty of Life Sciences and Education University of South Wales Pontypridd UK

**Keywords:** autoregulation, mean flow index, methodology, Mx, reliability

## Abstract

**Background:**

Mean flow index (Mxa) for evaluating dynamic cerebral autoregulation is derived using varying approaches for calculation, which may explain that the reliability ranges from poor to excellent. The comparability, repeatability, stability, and internal consistency of approaches have not previously been assessed.

**Methods:**

We included 60 recordings from resting healthy volunteers and calculated Mxa using four different approaches: three without overlapping calculations, using intervals for averaging wave‐form data (blocks) of 3, 6, and 10 s, and correlation periods (epochs) of 60, 240, and 300 s (3–60–F, 6–240–F, and 10–300–F); and one using 10‐second blocks, 300 s epochs, and overlaps of 60 s (10–300–60). The comparability between the approaches was assessed using Student's *t* test, intraclass correlation coefficients (ICC), and Bland–Altman plot.

**Results:**

Overall, 3–60–F resulted in a higher Mxa than the other indices (*p* < 0.001, for all). The reliability when comparing all the approaches ranged from moderate to good (ICC: 0.68; 95%CI: 0.59–0.84), which was primarily due to similarities between 10–300–F and 10–300–60 (ICC: 0.94; 95%CI: 0.86–0.98). The reliability when comparing the first and last half was poor for 10–300–F and ranged from poor to moderate for the other approaches. Additional random artifacts resulted in poor reliability for 10–300–F, while the other approaches were more stable.

**Conclusions:**

Mxa in general has a low sensitivity to artifacts, but otherwise seems highly dependent on the approach, with a repeatability that is moderate at best. The varying accuracy and precision renders Mxa unreliable for classifying impaired cerebral autoregulation when using healthy adults for comparison.

## Graphical abstract

Mx has a low sensitivity to artifacts, but otherwise seems highly dependent on the approach, with a repeatability that is moderate at best. The varying accuracy and precision renders Mx unreliable for classifying impaired cerebral autoregulation using healthy adults for comparison.

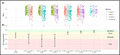

## 1 INTRODUCTION

Dynamic cerebral autoregulation is a physiological mechanism that serves to dampen changes in cerebral blood flow (CBF) secondary to acute fluctuations in cerebral perfusion pressures (CPP) through compensatory adjustments in cerebrovascular resistance (Strandgaard & Paulson, 1984). It may be assessed in humans through a wide array of transcranial Doppler ultrasound (TCD)‐based methods, of which the mean flow index (Mx) was introduced by Czosnyka et al., 1996. Mx was initially calculated as a correlation coefficient between CPP and middle cerebral artery velocity (MCAv) (Czosnyka et al., 1996). As an alternative approach, arterial blood pressure (ABP), measured invasively or noninvasively, has replaced ICP in patients and healthy volunteers where the latter is not readily available for the determination of CPP; the resulting measure is then typically coined Mxa (Zeiler et al., 2017). Mx and Mxa range from −1 to 1; high values are interpreted as inefficient dynamic cerebral autoregulation, and vice versa for low values. The most commonly used threshold for preserved versus impaired cerebral autoregulation is 0.3 (Czosnyka et al., 1996).

The reliability of Mxa has previously been assessed in healthy volunteers in several studies, which have reported highly variable repeatability and reproducibility ranging from poor to excellent (Chi et al., 2018; Lee et al., 2020; Lorenz et al., 2007; Mahdi, Nikolic, Birch, Olufsen, et al., 2017), and from poor to good (Lorenz et al., 2008; Ortega‐Gutierrez et al., 2014; Riberholt et al., 2021), respectively. As a potential explanation, these studies utilized short recordings, often shorter than 6 min, the minimum duration necessary for Mxa to stabilize according to one study (Mahdi et al., 2017). There are, furthermore, substantial differences in the approaches used to derive Mxa in the different studies, and there is currently no consensus on how to derive the most reliable value.

In the present study, we sought to assess the reliability of Mxa in resting healthy volunteers by measuring repeatability, stability, and internal consistency when exposing the same dataset to four different widely used approaches, with varying length of blocks, epochs, and recording length, and with the introduction of random artifacts.

## 2 METHODS

### 2.1 Ethical approval

The present work is based on data from four studies, previously published elsewhere (Berg et al., 2012, 2013; Riberholt et al., 2016, 2021), which were all approved by either the Scientific‐Ethical Committee of Copenhagen and Frederiksberg Municipalities (file numbers H‐A‐2009–020 and H‐2–2010–04) or the Regional Ethical Committee of the Capital Region of Copenhagen (file numbers H‐3–2013–024 and H‐16042103), and conformed to the standards set by the Declaration of Helsinki. No new ethical approval was necessary to conduct the present retrospective study. All subjects provided oral and written informed consent prior to inclusion. This study describes novel analyses of selected data from these studies to address an independent working hypothesis. The data and analyses that support the findings of this study can be shared upon reasonable request by contact to the corresponding author of this study and the original studies.

### 2.2 Subjects and recordings

This study encompasses recordings from a total of 48 healthy volunteers, with 62 individual baseline periods, which was defined as periods before any interventions were initiated. Subject and recording characteristics are provided in Table 1.

**TABLE 1:** Study characteristics

	Study A (*n* = 9)	Study B (*n* = 10)	Study C (*n* = 15)	Study D (*n* = 14)	All (*n* = 48)
Age – years ±SD	23 ± 2	23 ± 2	31 ± 13	28 ± 9	27 ± 9
Male – n (%)	9 (100%)	10 (100%)	7 (47%)	5 (36%)	31 (65%)
Recordings – n	9	10	15	28	62
Recording length – min ±SD	20.0 ± 1.8	17.9 ± 1.8	4.9 ± 0.4	5.2 ± 0.2	9.3 ± 6.5
Recordings longer than 15 min – n	9	10	0	0	19
Heart rate – min^−1^ ±SD	60 ± 9	58 ± 10	62 ± 8	63 ± 9	61 ± 9
Mean arterial pressure – mmHg ±SD	88 ± 6	84 ± 4	76 ± 13	66 ± 9	75 ± 12
Middle cerebral artery velocity – ^cm^/s ±SD	68 ± 11	71 ± 12	64 ± 18	75 ± 10	71 ± 13
Artifacts percentage – median (IQR)	0.1 (0–0.4)	0.5 (0.1–2.4)	0.1 (0–2.6)	2.2 (0.1–5.6)	0.45 (0–4.4)
Approach	Mxa	Mxa	nMxa	nMxa	—
3–60–F – mean ±SD	0.44 ± 0.15	0.58 ± 0.11	0.51 ± 0.15	0.32 ± 0.12	0.43 ± 0.16
6–240–F – mean ±SD	0.38 ± 0.15	0.48 ± 0.11	0.39 ± 0.28	0.22 ± 0.20	0.33 ± 0.23
10–300–F – mean ±SD	0.35 ± 0.18	0.45 ± 0.14	0.36 ± 0.29	0.17 ± 0.22	0.29 ± 0.25
10–300–60 – mean ±SD	0.36 ± 0.16	0.44 ± 0.15	0.38 ± 0.30	0.17 ± 0.19	0.29 ± 0.24

Abbreviation: nMxa, ABP is measured noninvasively.

### 2.3 Data collection

*Studies A* and *B* recorded invasive ABP in the left radial artery and MCAv by TCD insonation in healthy volunteers while lying supine with a slight elevation of the head (20°) (Berg et al., 2012, 2013). *Studies C* and *D* recorded ABP noninvasively with photopletysmographic continuous beat‐to‐beat measurement, and MCAv measured by TCD in the healthy volunteers while lying supine without head elevation (Riberholt et al., 2016). *Study D* recorded the same healthy volunteers twice separated by an interval of 23 ± 3 (mean, SD) days (Riberholt et al., 2021). Further details on data collection are described in full in the original publications.

### 2.4 Data processing

The recordings were extracted from LabChart into a tab‐delimited file in the original resolution of 1,000 Hz and visually inspected for artifacts. The artifacts were deleted by removing a period that started and ended in a curve nadir. To ensure sufficient quality of the calculations, blocks were omitted from the analysis if 50% of the raw measurements were missing, and similarly epochs were omitted if more than 50% of the blocks were missing. Mxa or nMxa was calculated using the clinmon function from the publicly available R package “clintools” v. 0.8.0 (Olsen & Riberholt, 2021).

### 2.5 Assessment of reliability

Reliability of Mxa and nMxa was assessed by comparing four different approaches, which pragmatically were chosen as the four most common approaches in the literature (Riberholt et al., 2021), here designated *3*–*60*–*F*, *6*–*240*–*F*, *10*–*300*–*F*, and *10*–*300*–*60*. In 3–60–F, 3‐second blocks and 60‐second epochs, that is, 20 blocks in every epoch, without overlaps were used; while 6‐second blocks and 240‐second epochs without overlaps were used in 6–240–F, 10‐second blocks and 300‐second epochs without overlaps were used in 10–300–F, and 10‐second blocks and 300‐second epochs with 60‐second overlaps were used in 10–300–60. Only recordings longer than 15 min were used to compare 10–300–F and 10–300–60, since shorter recordings would not “activate” the overlapping feature in 10–300–60.

**FIGURE 1:**
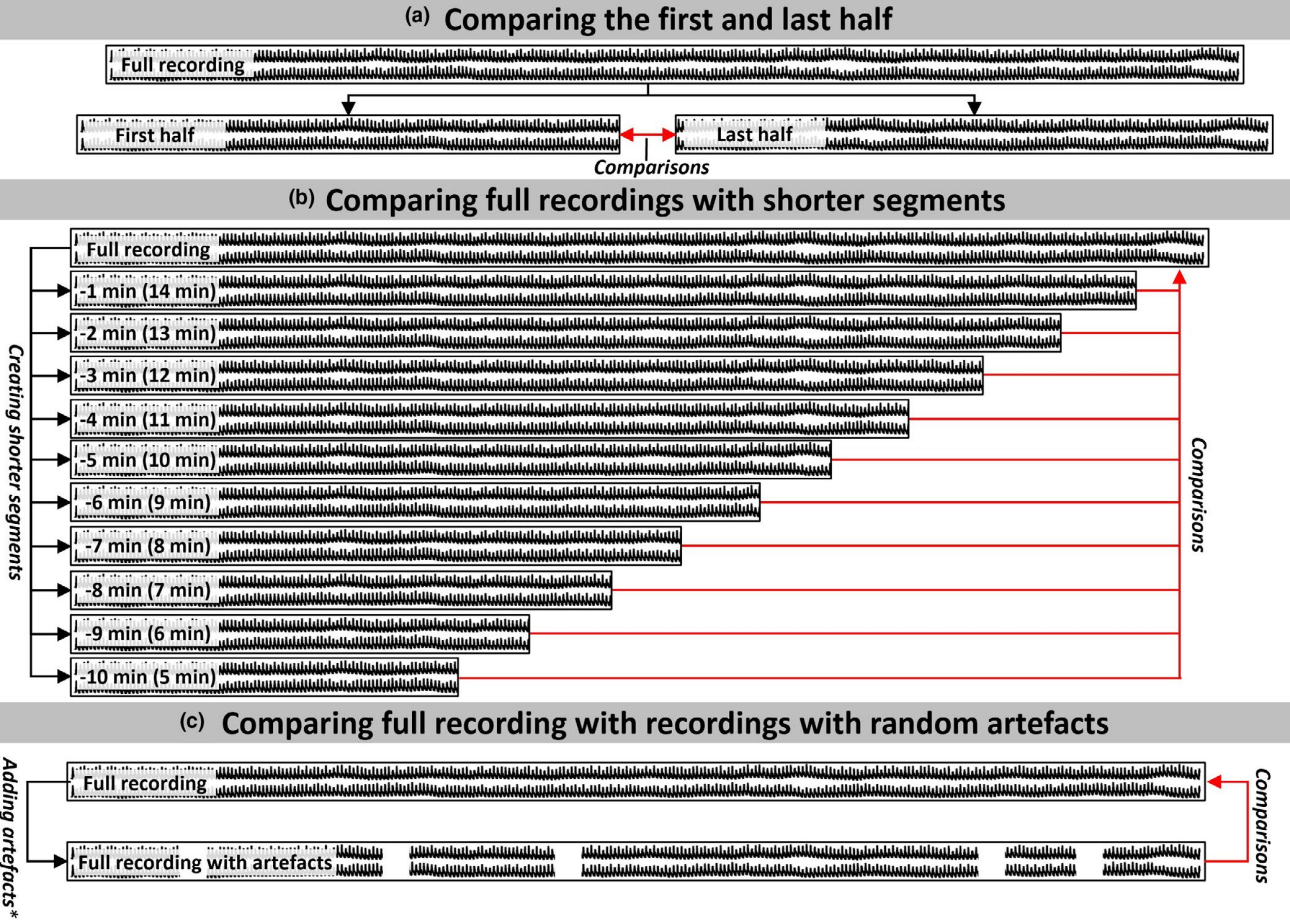
The approaches for assessing reliability were a comparison between (a) the first and last half of a recording; (b) comparing the full recordings with shorter segments of the same recording; and (c) the full recording and the same recording with random artifacts. The red arrows depict how the comparisons were carried out. * We calculated the addition of artifacts of varying length and percentage using 100 random artifact‐periods for each recording and chose the median Mxa‐value generated for comparison

For each of these approaches, repeatability was measured by comparing the first with the last half of recordings (Figure 1A), and by comparing recordings longer than 15 min with shorter segments of the same recording (Figure 1B). The latter was simulated by consecutively comparing the result from the full 15‐minutes with that of the same recording with a 1‐minute shorter duration (always removing the excess recording from the end), which was then repeated until recording length was 5 min.

The stability was assessed by introducing random artifacts of varying length (1–5 s) occupying a varying percentage (5%–50%) of the recording (Figure 1C). During these analyses, the quality restrictions in percentage available data, described above, was ignored. Each recording underwent one hundred imputations with randomly deleted periods for each artifact, length, and percentage of the total recording. Manually identified artifacts were always deleted before analysis, since inclusion of those in the analysis would introduce further bias.

### 2.6 Statistical analysis

All statistical analyses were carried out using R 4.0.2 (R Core Team (2020), Vienna, Austria). If not specified, normally distributed data are presented as mean (±SD), while non‐normally distributed data are presented as median (IQR). Paired Student's *t* test was applied to compare groups, and p values are presented after Bonferroni correction. Reliability was calculated using the two‐way mixed‐effects single measurement absolute agreement intraclass correlation coefficient (ICC), and classified as poor (<0.5), moderate (0.5–0.75), good (0.75–0.9), or excellent (>0.9) with reference to both the lower and upper confidence limits (Koo & Li, 2016). Furthermore, Bland–Altman plots with the limits of agreement (LOA) were generated to quantify differences (Bland & Altman, 1986). Error bars in the figures represent the 95% confidence interval (95%CI).

## 3 RESULTS

**FIGURE 2:**
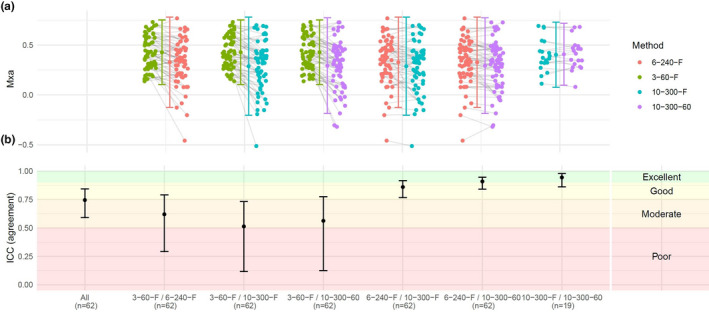
Comparison between the same recording using different approaches. (a) The recording assessed with different approaches showing the Mxa for every participant, with gray lines depicting the relationship between the results gained from the left and right approach for each comparison. (b) The ICC when comparison all approaches, and between each. ICC, Intraclass correlation coefficient

Overall, 3–60–F resulted in a higher Mxa than the other approaches (*p* < 0.001, for all), while 6–240–F yielded a higher Mxa than 10–300–60 (*p* = 0.03), and the Mxa resulting from 10–300–F did not differ significantly from that of 10–300–60 or 6–240–F (Figure 2A). The reliability when comparing all the approaches ranged from moderate to good (ICC: 0.68; 95%CI: 0.59 to 0.84), which could be primarily credited to the similarities between 10–300–F and 10–300–60 (ICC: 0.94; 95%CI: 0.86 to 0.98) (Figure 2B). This similarity was also reflected in the Bland–Altman plot, which showed almost no systematic bias when 10–300–F and 10–300–60 were compared (bias: 0.01; LOA: −0.16 to 0.17). Comparison of 3–60–F with 10–300–F (bias: 0.14; LOA: −0.21 to 0.49) and 3–60–F with 10–300–60 (bias: 0.13; LOA: −0.19 to 0.45) resulted in wider LOA and a systematic bias with 3–60–F being higher in general. Similarly, 6–240–F was higher than 10–300–F (bias: 0.04; LOA: −0.20 to 0.28) and 10–300–60 (bias: 0.03; LOA: −0.15 to 0.22), but lower than 3–60–F (bias: −0.10; LOA: −0.40 to 0.20) (Figure S1).

**FIGURE 3:**
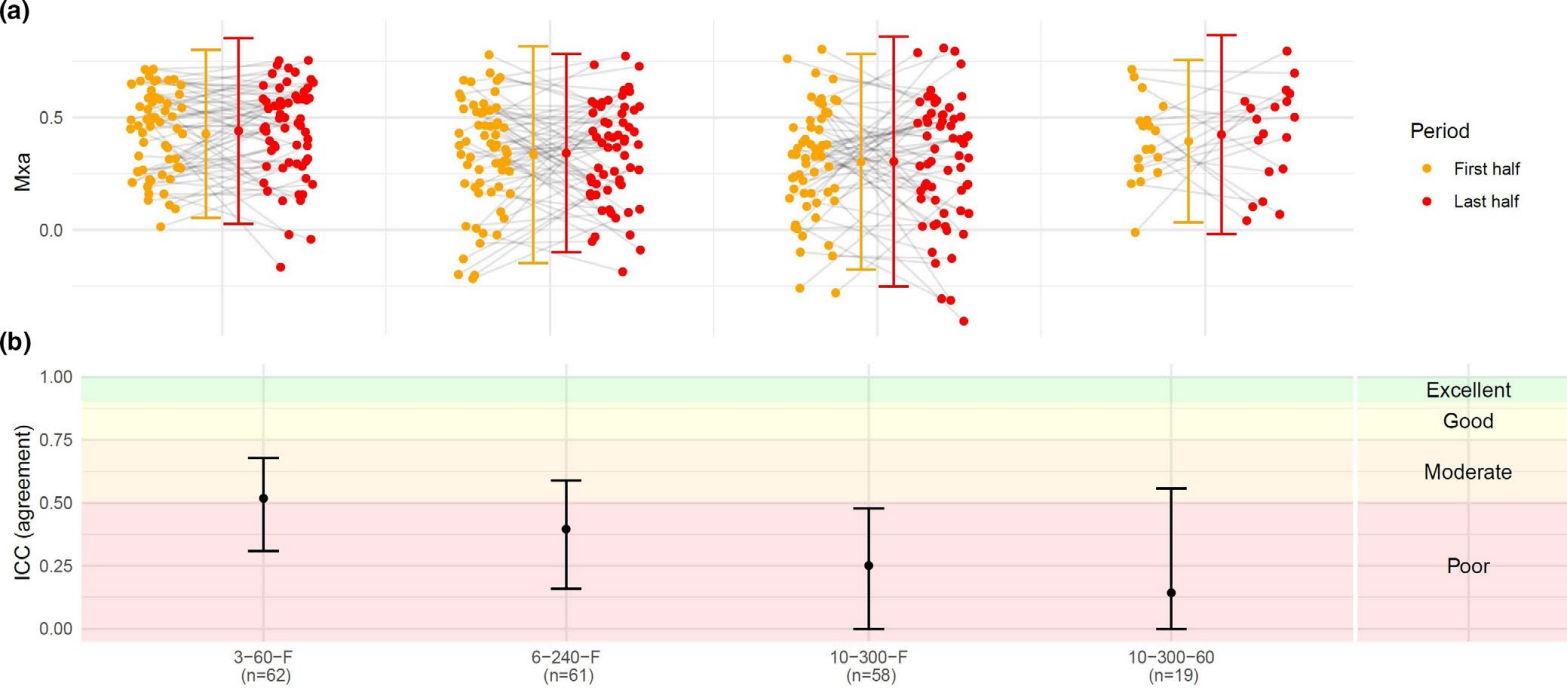
Comparison between the first and last half of a recording with different approaches. (a) The Mxa for the first and last half of the recordings, with grey lines depicting the relationship between the results gained from the first and last half. Only recordings with at least two epochs were included in analysis of 10–300–60, that is a duration of more than 6 min (*n*=19). (b) The ICC for each approach. ICC, Intraclass correlation coefficient

The four approaches showed similar mean and standard deviation when comparing the first and last half of the recordings (first vs. last; 3–60–F: 0.43 ±0.19 vs. 0.44 ±0.21; 6–240–F: 0.34 ±0.24 vs. 0.34 ±0.22; 10–300–F: 0.30 ±0.24 vs. 0.30 ±0.28; 10–300–60: 0.39 ±0.18; 0.42 ±0.22) (Figure 3A). The reliability ranged from poor to moderate for 3–60–F (ICC: 0.52; 95%CI: 0.31 to 0.68), 6–240–F (ICC: 0.40; 95%CI: 0.16 to 0.59), and 10–300–60 (ICC: 0.14; 95%CI: −0.34 to 0.56), and was poor for 10–300–F (ICC: 0.25; 95%CI: −0.01 to 0.48) (Figure 3B). The narrowest LOA was found with 3–60–F (3–60–F, bias: −0.01; LOA: −0.39 to 0.37; 6–240–F, bias: −0.01; LOA: −0.51 to 0.49; 10–300–F, bias: −0.01; LOA: −0.64 to 0.62; 10–300–60, bias: −0.03; LOA: −0.55 to 0.49) (Figure S2).

**FIGURE 4:**
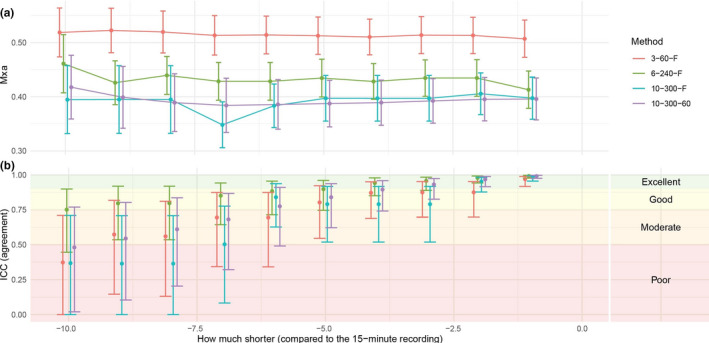
Comparison between the full 15‐minutes and shorter segments of the same recording for each approach (colors). The figures presents (a) the Mxa for the recordings of different lengths; (b) The ICC for each approach (colors) and for each segment which is compared to the full 15‐minutes. ICC, Intraclass correlation coefficient

Mxa calculated from 15‐minute recordings (*n* = 18; 3–60–F: 0.51 ±0.15; 10–300–F: 0.40 ±0.16; 10–300–60: 0.40 ±0.16) did not differ from that of the shorter recordings (Figure 4A). The reliability was good to excellent when comparing the first 13 and 14 min of the recordings with the full 15 min for all three approaches, while 10–300–F and 10–300–60 showed poor to good reliability when including nine minutes or less to compare with the full 15 min (Figure 4B). The absolute difference between the full 15 min and the shorter recording decreased when increasing the recording length of the comparator (Figure S3).

**FIGURE 5:**
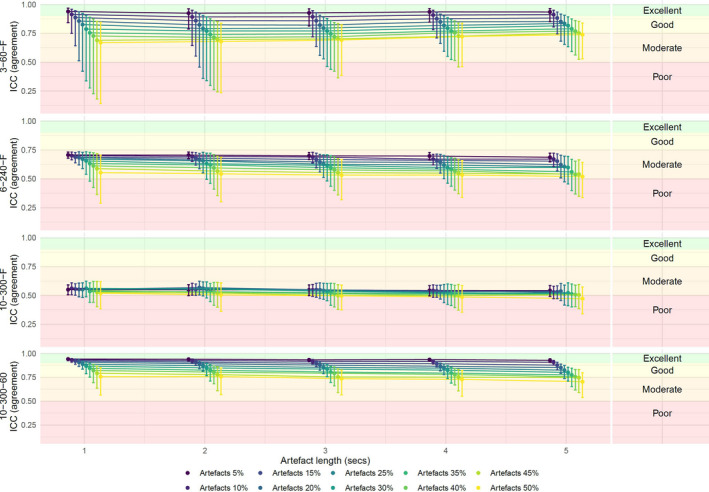
The ICC for each approach when comparing artifacts with a length between 1 and 5 s (x‐axis), and between 5% and 50% of the recording (colors). ICC, Intraclass correlation coefficient

The addition of artifacts without quality control showed that increasing percentage and length of artifacts lowered the reliability for all the approaches. Overall, any additional artifacts resulted in poor reliability for 10–300–F; for 6–240–F and 3–60–F, respectively, poor reliability was identified after the addition of 25% and 40% artifacts. 10–300–60 was more robust and together with 3–60–F showed excellent reliability after the addition of 5% artifacts. 6–240–F and 10–300–F showed moderate reliability at best, when only 5% of artifacts were added (Figure 5).

## 4 DISCUSSION

The findings of this study highlight that a given Mxa value depends greatly on the methodological details, including the length of blocks and epochs. This is the first study to compare values of Mxa resulting from different approaches; although this measure appears to be robust towards artifacts, other of our findings question its reliability. The healthy volunteers had an average Mxa close to the usual threshold for impaired cerebral autoregulation of 0.3, which is somewhat high, but comparable to previous reports (Ortega‐Gutierrez et al., 2014; Reinhard et al., 2007; Yam et al., 2005).

In this study, we compared four commonly used approaches to data collection and calculation. Although reliability was good to excellent for comparisons between three of the approaches (6–240–F, 10–300–F and 10–300–60), it deteriorated to a result reliability between poor and good for comparison with 3–60–F, which is the second most widely used approach in the literature. The findings indicate that Mxa is strongly influenced by changes in the length of blocks and epochs, and that comparison of Mxa between studies with different methodology is problematic. This issue is also reflected in the substantial bias with wide LOA in Bland–Altman plots. 3–60–F, in general, resulted in higher Mxa values than other approaches; more than 50% of measurements in healthy volunteers (who should exhibit intact autoregulation) were higher than 0.30, a commonly applied threshold for identifying impaired cerebral autoregulation (Altamura et al., 2009; Czosnyka et al., 2003; Kermorgant et al., 2019; Mahdi, Nikolic, Birch, & Payne, 2017; Nasr et al., 2011, 2014; Schmidt et al., 2003). One possible explanation for the higher Mxa in 3–60–F is that each 3‐second block is affected by respiratory waves, and that the impact of this is lessened when longer block sizes are used (Czosnyka et al., 2003). Even though 3–60–F resulted in the highest Mxa, dichotomization between intact and impaired cerebral autoregulation in the other approaches still seem inappropriate. This difference between 3–60–F and the other approaches questions both if the estimate of cerebral autoregulation is comparable, and maybe more important if studies which utilize different approaches are comparable.

Previous studies have assessed the repeatability by comparing the first and last half of recordings, reporting poor to moderate repeatability (Lorenz et al., 2007, 2008). This pattern applies to all approaches in the present study. As an exception from the rule, one previous study showed excellent repeatability of Mxa when the first or last half of a recording was compared with the full recording of 10 min (Chi et al., 2018). This excellent reliability when comparing overlapping segments, is only reproduced in our data when comparing 14‐ with the full 15‐minute recording. Across approaches, a marked reduction in reliability is observed at 9 min, and at 5 min the reliability of all approaches is poor. 3–60–F presents the best overall reliability for all recording lengths, which corresponds to simply removing one epoch for every minute the recording is shortened. This stresses that a higher number of epochs for the same recording increases the stability of Mxa. 3–60–F seems the least susceptible to variations in shorter recordings, which primarily is due to the shorter epochs, why utilization of 6–240–F, 10–300–F, 10–300–60 is only recommended when using substantially longer recordings. Our findings of poor to moderate repeatability is comparable to previous reports of other indices for dynamic cerebral autoregulation, including index of autoregulation and transfer functions analysis (Brodie et al., 2009; Gommer et al., 2010).

The stability of Mxa assessed when adding random artifacts shows decreasing reliability with the best reliability for 3–60–F and 10–300–60. The length and number of artifacts did not seem to affect 10–300–F as much as the three other approaches, which exhibited poor reliability even after adding only 5% artifacts. The number of blocks and epochs seems to be an important factor for reliability for Mxa.

The internal consistency refers to the stability of Mxa on a group level and ignores the individual variations (Bannigan & Watson, 2009). The internal consistency of Mxa is primarily related to the length of blocks and epochs. In contrast, the recording duration and amount of artifacts appear to be less critical.

### 4.1 Strength and limitations

The main strength of this study is the use of clinically relevant data and strict criteria for assessing reliability defined as repeatability, stability, and internal consistency. Since the data were collected for another purpose unintentional confounder might be present. We did not include all the approaches described in the literature for this analysis, but nonetheless believe that the chosen examples underline the influence of details in the approach used to generate Mxa. As another limitation, the variation in recording length between the studies pooled in this study may have affected some of the reliability measures. Finally, this study was designed neither to interpret the clinical relevance nor the difference between groups of Mxa in clinical studies.

## 5 CONCLUSION

According to the present findings, the reliability of Mx, in our example Mxa, as a generic index is questionable. While being relatively insensitive to artifacts, the calculation of Mxa is highly dependent on the underlying approach, notably recording length, and the length and number of blocks and epochs. We suggest that caution is warranted for the comparison of Mxa reported by different studies. The varying accuracy and precision, furthermore, renders Mxa unreliable for classifying impaired cerebral autoregulation using healthy adults for comparison.

## CONFLICT OF INTEREST

The authors have no conflict of interest to report.

## AUTHOR CONTRIBUTIONS

MHO, CR, KM, and RMGB designed the study; CR, RRP, and RMGB collected the data; MHO did the analyses and wrote first draft; all authors revised and approved final version.

## Supporting information



Fig S1

Fig S2

Fig S3
