# Supplementary figures and images for: Reliability of the mean flow index (Mx) for assessing cerebral autoregulation in healthy volunteers
Source: Physiol Rep. 2021 Jun 26;9(12):e14923. doi: 10.14814/phy2.14923 (PMC8234479; doi:10.14814/phy2.14923)

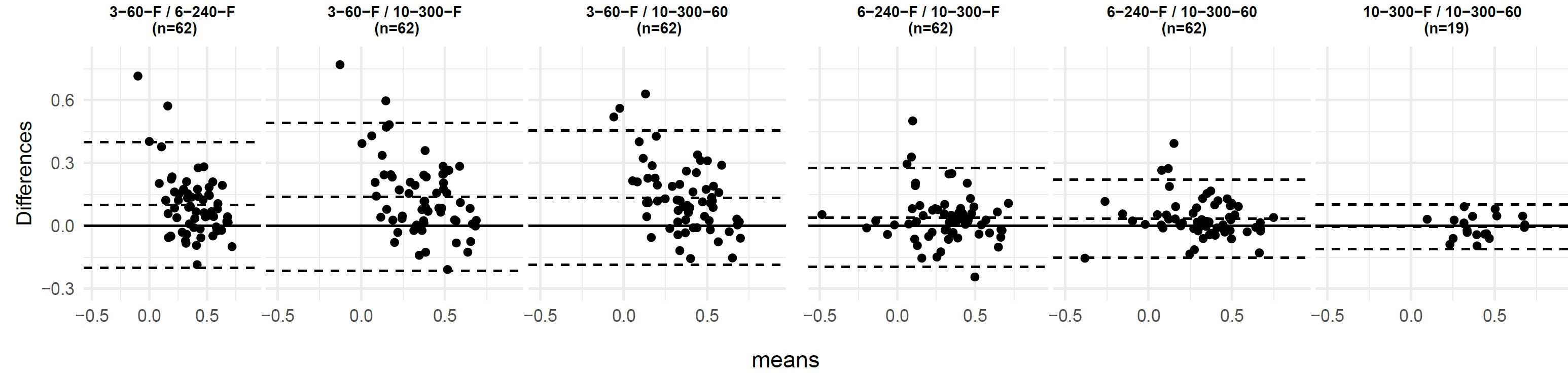

Supplement: Supplementary file 1 — Fig S1 [file PHY2-9-e14923-s003.JPG]

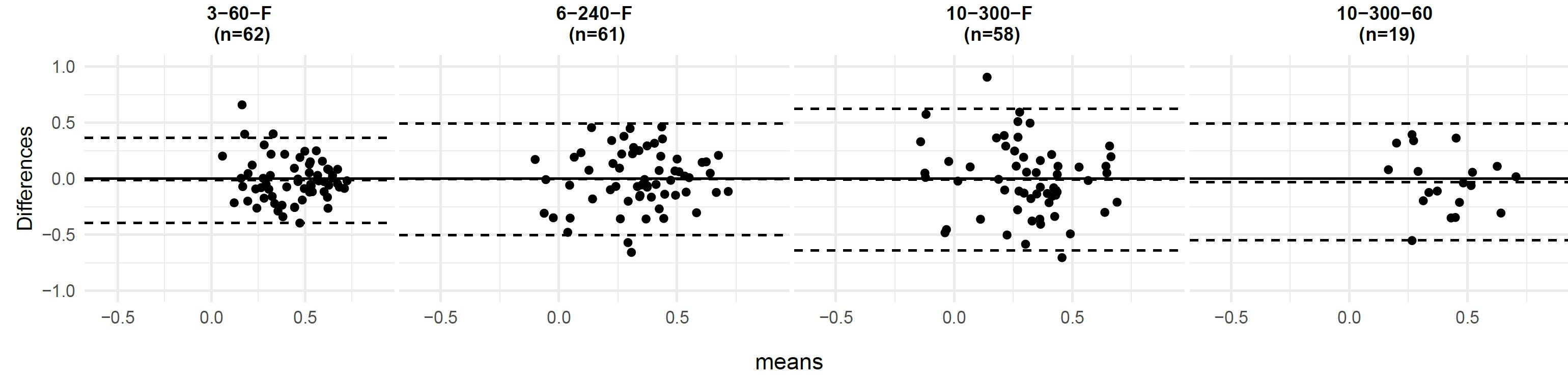

Supplement: Supplementary file 2 — Fig S2 [file PHY2-9-e14923-s002.JPG]

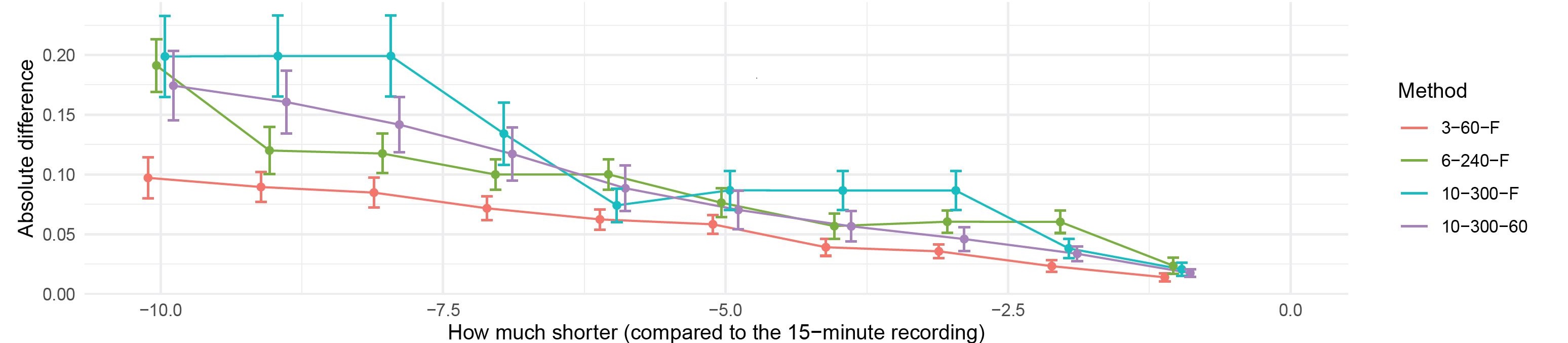

Supplement: Supplementary file 3 — Fig S3 [file PHY2-9-e14923-s001.JPG]
